# Selective Content Retrieval in Information-Centric Networking

**DOI:** 10.3390/s22228742

**Published:** 2022-11-12

**Authors:** José Quevedo, Daniel Corujo

**Affiliations:** 1 Instituto de Telecomunicações, 3810-193 Aveiro, Portugal; 2 Departamento de Electrónica, Telecomunicações e Informática, Universidade de Aveiro, 3810-193 Aveiro, Portugal

**Keywords:** Internet of Things, information-centric networking, in-network processing

## Abstract

Recently, novel networking architectures have emerged to cope with the fast-evolving and new Internet utilisation patterns. Information-Centric Networking (ICN) is a prominent example of this architecture. By perceiving content as the core element of the networking functionalities, ICN opens up a whole new avenue of information exchange optimisation possibilities. This paper presents an approach that progresses the base operation of ICN and leverages content identification right at the network layer, allowing to selectively retrieve partial pieces of information from content already present in ICN in-network caches. Additionally, this proposal enables information producers to seamlessly offload some content processing tasks into the network. The concept is discussed and demonstrated through a proof-of-concept prototype targeting an Internet of Things (IoT) scenario, where consumers retrieve specific pieces of the whole information generated by sensors. The obtained results showcase reduced traffic and storage consumption at the core of the network.

## 1. Introduction

Over the years, we have witnessed the evolution of the Internet from its original purpose of interconnecting a reduced set of computers, to the provisioning of worldwide connectivity to more than 18 billion devices [1]. Nowadays, users are mainly interested in retrieving content and accessing services regardless of the location where they are being provided. New networking technologies have been emerging to exploit such utilisation scenarios. A prominent example lies with Information-Centric Networking (ICN) [2,3], which centres its networking functionalities around the content itself, moving away from the host-centric approach followed in current IP-based network deployments.

Parallel to this, the advances in sensor technologies have promoted the emergence of new usage scenarios under the umbrella of the Internet of Things (IoT) [4,5,6,7]. The IoT features one of the most heterogeneous type of environments, considering devices with different capabilities in terms of communication and processing resources, as well as with different ways of information representation. Additionally, IoT has been one of the many cases which have manifested high adoption capability of new technologies, such as leveraging massive Machine Type Communications (mMTC) capabilities in scenarios featuring deployments of the Fifth generation of mobile communications (5G) [8]. Consequently, it has placed a new set of stringent requirements over the underlying networking fabric, creating the need for novel ideas and solutions able to address such requirements while standing to the trial of further upcoming challenges [9].

Furthermore, the suitability of the TCP/IP stack for supporting IoT has been questioned [10], creating an appealing opportunity for the assessment of ICN as a key element in a simplified architecture towards IoT interaction. This opportunity has encouraged the utilisation of ICN concepts for addressing IoT challenges [11,12,13]. Notably, the Information-Centric Networking Research Group (ICNRG) of the Internet Research Task Force (IRTF) has identified IoT as a baseline scenario where the use of ICN could bring significant advantages compared to existing Internet protocols [14]. Moreover, the deployment of current and envisioned IoT scenarios over ICN architectures can provide valuable insight into the key enablers and extensions to the ICN base design (e.g., multi-source data retrieval [15], enhanced producer mobility [16], and priority-based forwarding [17]). Accordingly, ICN will more holistically support fully-fledged Internet mechanism requirements, opening up a whole new set of scenarios that are not feasible under the current Internet architecture.

Different ICN implementations follow different design choices (e.g., communication model, naming principles, routing, and forwarding). This work focuses on Interest-based ICN solutions, particularly on the Named Data Networking (NDN) protocol [18]. This protocol envisions consumers that express their interest in retrieving named pieces of data. In this way, the name can be seen as a data selector, where the data is distributed over the network. The primary benefit of these protocols is the availability of network caching. However, granular caching is only available if the content producer publishes data with the desired granularity. As such, although content (dis)aggregation could largely fit IoT scenarios, it defeats the benefits of ICN caching. Moreover, a desirable consequence of distributing the content over the network is to offload the data provisioning work, yet there are no mechanisms for in-network content (dis)aggregation.

Thus, the contributions of the current paper are two-fold: (i) to provide the means for selectively accessing specific parts of a given content, enabling the (dis)aggregation of IoT content while still leveraging ICN in-network caching; and (ii) to enable an in-network processing approach that allows the offloading of the (dis)aggregation process to the network itself.

These contributions are materialised and assessed through the development of a proof-of-concept prototype following the NDN principles. The assessment is performed through simulation, allowing to extend the scope of the evaluation to a larger scale.

**Figure 1:**
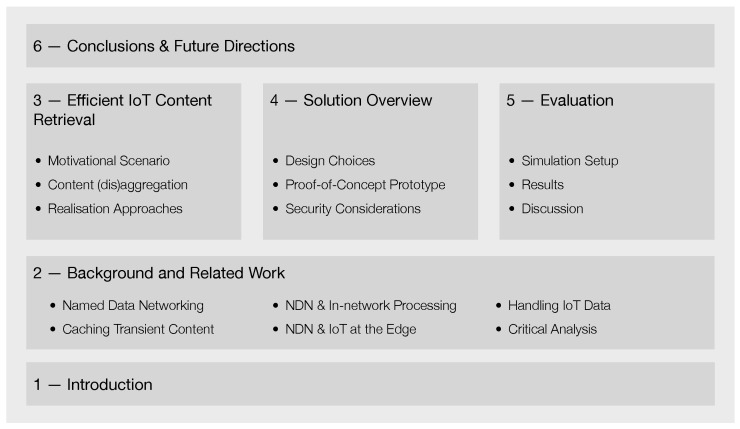
Paper organization.

The remainder of this paper is structured as presented in Figure 1. After this introduction, Section 2 briefly introduces NDN foundations and provides an overview of previous work relevant in the context of the paper. Section 3 exposes the motivation for the development of this work and discusses strategies for approaching the problem. Section 4 details the proposed solution and Section 5 provides the assessment of the solution by presenting and discussing the evaluation results. Finally, the conclusions are drawn in Section 6.

## 2. Background and Related Work

### 2.1. Named Data Networking

In NDN, the communication process is initiated by consumers, which send a request (i.e., an Interest packet). This packet is forwarded by intermediary nodes towards an entity holding the content, according to the information stored in the Forwarding Information Base (FIB) and following a configured forwarding strategy. The network nodes maintain a Pending Interest Table (PIT) with information about the forwarded requests. Subsequent requests for the same content are aggregated in the PIT. Responses (i.e., data packets) follow the reverse request path based on the state information stored in the PITs. When a node forwards a data packet, the Interest is considered to be satisfied, and the corresponding PIT entries are removed. Content objects are signed by the producers, ensuring both integrity and authenticity of the content and can be cached in the intermediary Content Stores (CS).

### 2.2. Caching Transient Content in NDN

In fact, the NDN caching mechanism has a prominent role in providing an increased data delivery performance and has been identified as a key enabler towards a name-based IoT [19]. However, in IoT scenarios, new information is constantly being generated and consumers are mainly interested in the latest information (i.e., transient data). To address this issue, NDN includes an optional *FreshnessPeriod* parameter in data packets to indicate how long a certain packet could be held in the network content stores before marking it as “non-fresh”. While “non-fresh” data is still valid, it is an indication that the producer may have issued newer data.

The caching of transient IoT content has been explored in different stages of the caching process: (i) caching strategies considering the popularity of IoT data and their lifetime [20,21]; (ii) caching replacement policies [22]; and retrieval of fresh content [23].

### 2.3. NDN and IoT Services at the Edge

The integration of edge computing and networking solutions has emerged as a promising technology with the potential to impact the different elements of an end-to-end system (i.e., producers, consumers, and the subjacent networking fabric). The intrinsic capabilities of NDN makes it a suitable candidate for joint edge computing and communication solutions, whereas IoT stands out as one of the scenarios that could better profit edge approaches.

In particular, NDN has been explored for processing IoT data at the edge, thus reducing the latency perceived by the users and the network overhead that would be required for unnecessary transport of raw IoT through the network core [24,25]. Edge has also been explored for fulfilling tasks that would otherwise not be feasible in constrained devices (e.g., caching and forwarding tables [26], security [27], mobility [28], and keywords-based named schemes [29]).

### 2.4. In-Network Processing via NDN

In addition to edge computing, in-network processing has been increasingly gaining significance, empowered by the evolution toward a programmable data plane [30] and further leveraging NDN principles towards what has been denoted as Named Function Networking (NFN) [31,32]. NFN extends NDN to support the on-demand execution of λ-functions using networking resources to satisfy complex user requests. In this context, NDN forwarding strategies play a prominent role in the execution performance of this distributed computation approach [33]. Moreover, the NFN concept has been extended towards the edge of the network to better accommodate IoT scenarios [34,35]. Furthermore, NFN has been also applied for enhanced content reachability [36]; the resulting NFN-based structured query language is considered to be an initial step towards the network-based content (dis)aggregation.

In addition, with the goal of bringing computation closer to consumers and reduce the usage of network resources while providing better latency, Named Function as Service (NFaaS) [37] takes a step forward in the execution of named functions and imports the concept of serverless architectures into the NDN domain. In the same line, RICE [38], a fundamental building part of the Computation First Networking (CFN) solution [39], enables the invocation of remote methods towards the provisioning of a distributed computing framework. These solutions take into consideration the possibility of executing long-running computations by decoupling the method invocation from the result retrieval.

### 2.5. Handling IoT Data

The aggregation of IoT content (as a way to save scarce resources and reduce traffic load) has been the focus of numerous research works [40]. Different aggregation techniques have been proposed impacting the performance of the network in terms of latency, throughput, and energy consumption, among others [41]. In ICN, a fundamental aspect of data aggregation for the IoT is related to the need of retrieving information from multiple sources [15] (e.g., gather the weight values from all the sensors in the wheels of a train composition). This concept is often combined with in-network processing for more advanced features [42] (e.g., retrieve the average weight carried over a train car).

Novel ICN-based frameworks have been proposed to handle IoT data with increased focus on specific criteria (e.g., security [43], flexible content retrieval [44], and data communication efficiency [45].

### 2.6. Critical Summary

The potential impact of NDN in IoT has been acknowledged by the research community with an increasing interest in leveraging caching mechanisms and computation (e.g., edge and in-network processing) toward providing improved data delivery performance for constrained devices. Although some of the existing solutions share our motivation, to the best of our knowledge there is no prior work that targets the selective retrieval of content as part of the forwarding process by leveraging in-network computation. In this context, the solution proposed in this paper will provide an extendable and name-independent selective content retrieval mechanism that inherently supports function offloading, through a distributed in-network processing approach. Moreover, the proposed mechanism will do so while retaining the benefits associated to NDN’s intrinsic caching mechanisms.

## 3. Efficient IoT Content Retrieval

The current section uses a motivational scenario to showcase the need to support efficient IoT content retrieval by enhancing the basic features of the NDN architecture. Different considerations and approaches to realise these enhancements are discussed.

### 3.1. Motivational Scenario

In general, IoT devices provide different information, typically generating small pieces of content. As such, the aggregation of IoT information is a shared vision for IoT content dissemination optimisation. This raises the question of how the ICN functionalities can be better leveraged for the (dis)aggregation of IoT content.

**Figure 2:**
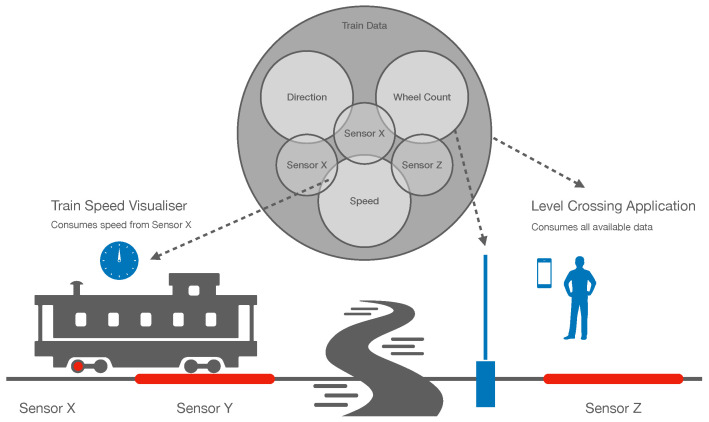
Motivational scenario: IoT data (dis)aggregation.

This question is the motivation for conducting this research. The foundations for the development of this work can be observed from Figure 2. The figure represents one of the scenarios of the H2020 5Growth project [8] where the information from different railroad train sensors (i.e., X, Y, and Z) is aggregated into more general content, such as “*Wheel Count*”, “*Direction*” and “*Speed*” (i.e., we aim to have a system that allows to seamlessly request the overall aggregated “*Speed*” of the train, or the current “*Speed*” value of independent sensors existing in a particular train car, or in a specific train wheel). The purpose of this information is to be sent through the network and absorbed by actuators, such as a railroad crossing controller that is able to lower or raise the road gate when an incoming train is detected. The scenario also considers different end-points that may be interested in different pieces of the aggregated content (e.g., the on-board speedometer consumes the speed of the train measured from the specific sensor X). The challenge ahead lies in how to enable network nodes to selectively retrieve these pieces of content from the intrinsic ICN in-network caching, reducing the extra network overhead and releasing the information producers (possibly with constrained resources) from the content processing tasks.

### 3.2. On NDN and Content (Dis)Aggregation

**Figure 3:**
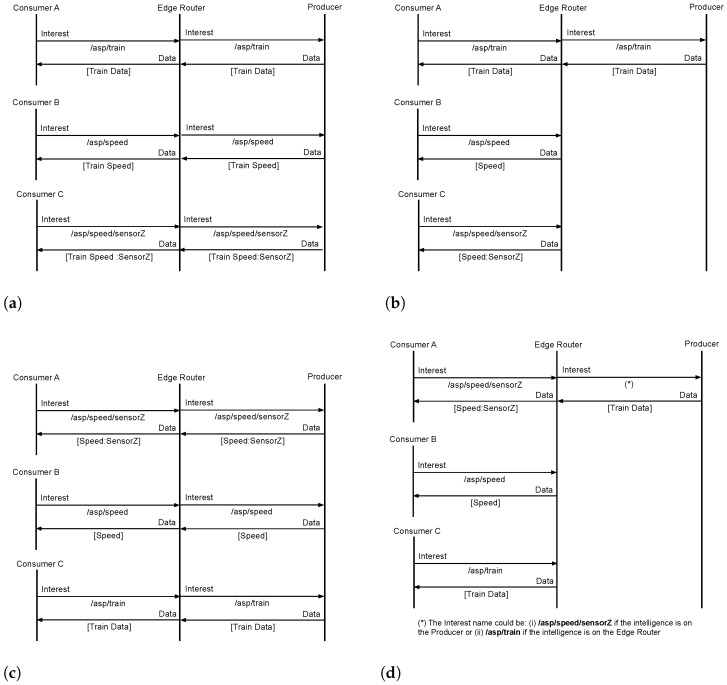
NDN strategies for selective content retrieval: (**a**) Simple Request; (**b**) Advanced Request; (**c**) Simple Response; (**d**) Advance Response.

To place the previous scenario into perspective, let us consider it from a basic NDN point of view, as shown in Figure 3a. First, *Consumer A* requests the aggregated content of the whole train (i.e., *Train Data*), which is not available in the intermediary node content store and has to be provided by the information producer. Next, Consumers *B* and *C* request *Train Speed* and *Train Speed from Sensor Z* contents, respectively. Although the actual information is already available at the *Edge Router* (i.e., as part of the larger *Weather* content), the said router does not have the knowledge to extract the desired information. Consequently, these requests have to be satisfied by the *Producer*.

To avoid the unnecessary forwarding of requests towards the source, it is possible to add some intelligence to intermediary routers, enabling them to extract the requested information from the aggregated data available in the content store. Such behaviour is shown in Figure 3b. In this case, the requests from Consumers *B* and *C* are satisfied by the edge router, which extracts the necessary information from the cached *Train Data* content.

Still, with such an enhancement, the order of arrival of the incoming requests at the intermediary routers also impacts the retrieval mechanism, as represented in Figure 3c. This time, Consumers *A* and *B* request partial pieces of the aggregated *Train Data* content. Since the content is not available in the cache, these requests, as well as the request from *Consumer C*, have to be satisfied by the *Producer*. As a consequence, *Speed from Sensor Z* information is retrieved three times (i.e., it is actually included in every data packet sent by the Producer) and the *Speed* information is retrieved twice (i.e., it is included in the last two data packets sent by the Producer).

This highlights the need for a more advanced response strategy, one that recognises that the piece of content being requested is part of a larger aggregated content and that preemptively pushes this larger content towards the edge of the network for later processing. Two different approaches are envisioned, as shown in Figure 3d, to fulfil such a purpose: (i) the intelligence is on the Producer: the *Edge Router* forwards the request for the smaller piece of content, and the producer replies with the larger aggregated content (i.e. *Train Data*); (ii) the intelligence is on the router: the *Edge Router* takes the decision of extending the request to a larger piece of content (/asp/train) and the Producer responds accordingly. Notwithstanding, these kinds of approaches require more detailed agreements between the producer and the network (e.g., how much level of aggregation should be pushed into the network without an explicit request?).

### 3.3. Realisation Approaches

**Figure 4:**
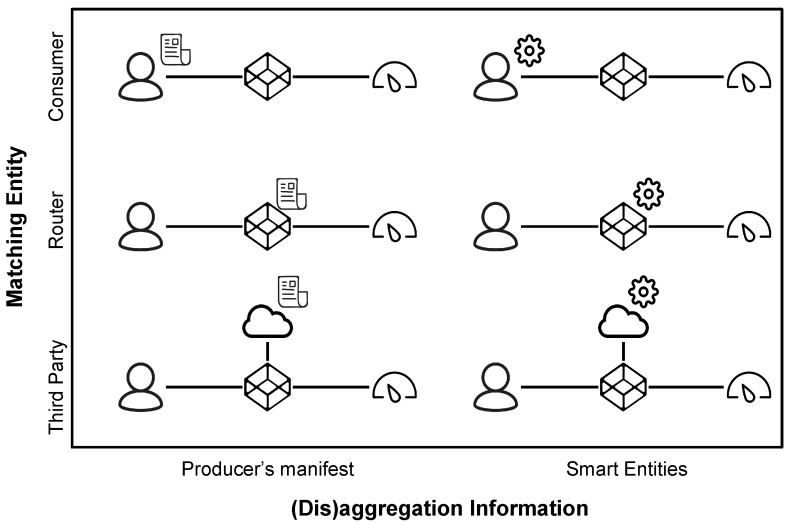
Dis(aggregation) strategies.

As it has been already established, the goal of this work is to provide network-assisted content (dis)aggregation in different scenarios, as befitting consumers, producers, or network operators. The balance between these three parties defines the possible targets of enhancements. As such, the current section explores different strategies for realising a mechanism that fulfils the vision presented in Section 3.2. For convenience, the proposed strategies are summarised in Figure 4.

According to the source of the (dis)aggregation information, the resulting strategies fall into two categories:

- The producer provides detailed content (dis)aggregation information (e.g., a manifest detailing the structure of the content and how to extract the different pieces listed in such a manifest);
- A network entity has the content (dis)aggregation intelligence (e.g., the network entity implements the extraction functionalities which are run based on the incoming requests).

Depending on the holder of the (dis)aggregation knowledge, the (dis)aggregation information strategies fall into four categories:

- Consumer: consumers hold the (dis)aggregation knowledge and generate complex queries that identify the desired piece of content. For such an approach to work, the intermediary routers must implement and support a (dis)aggregation protocol;
- Router: intermediary routers hold the (dis)aggregation knowledge and processes incoming Interests and return the associated data. Consumers should have the required knowledge to request pieces of content (e.g., through discovery procedures);
- Third party: a third party entity holds the (dis)aggregation information. In this approach, the (dis)aggregation duties are offloaded to the third party entity, which must implement a (dis)aggregation protocol known to users and/or routers.

Additionally, hybrid approaches in which the dis-aggregation knowledge is spread over different networking entities are also envisioned.

## 4. Solution Overview

After discussing the different strategies for enabling efficient IoT content retrieval, the current section presents a solution that falls into the “Smart Router” approach. Particularly, we propose to provide the NDN intermediary nodes (or at least those at the edge of the network) with the capabilities to execute queries on cached content, thus removing this burden from the producer of the information. This section explains the design choices that guided the development of the solution, provides the implementation details of the proof-of-concept prototype, and discusses the main security concerns associated with the proposed solution.

### 4.1. Design Choices

As discussed earlier, the proposed solution was deployed over the NDN networking architecture. In doing so, keeping the proposed enhancements as generic as possible without affecting the NDN design principles, is a major design requirement. The solution focuses on network-assisted dis-aggregation of content, leaving the generation of the aggregated content out of the scope of the proposal, and leading to what will be denoted as the Content-in-Content (CiC) retrieval mechanism. In realising this concept, the intelligence of the network will be placed on the forwarding nodes, particularly on those at the edge of the network. The goal is to allow the processing offloading from the likely constrained IoT sensors and to have the information closer to the end-user. As for the dis-aggregation process, for simplicity, it will be assumed that the content is structured and a query language could be used for extracting the desired part of the content. Notwithstanding, the proposed mechanism should be extendable to non-structured content, relying upon more advanced content extraction functionalities (e.g., retrieve only the faces from a live stream security surveillance feed by relying on video analytics). Finally, the solution should provide enhanced operations such as the ones described in Figure 3d, and must retain backward compatibility, ensuring its interoperability, enabling scenarios featuring both enhanced and basic routers.

### 4.2. Proof-of-Concept Prototype

The proof-of-concept prototype was developed using the NDN C++ library with eXperimental eXtensions (ndn-cxx) and the NDN Forwarding Daemon (NFD) implementations (version 0.6.5).

In our proposal, content selection queries are embedded into the Interest packets, using the *ApplicationParameters* field. This field can carry an arbitrary amount of information and is used to parametrise the request for Data. The intention of this field is to allow consumers to customise the Data they are willing to receive. As such, this field is processed by the information Producer. The purpose of this field matches the requirements of this work, and as such we propose to let intermediary nodes interpret and process it.

**Figure 5:**
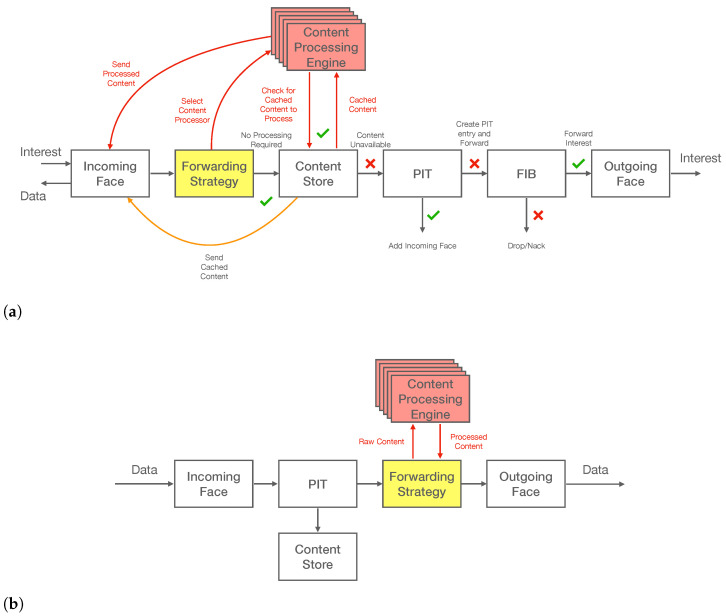
CiC forwarding: (**a**) Interest processing pipeline; (**b**) Data processing pipeline.

To provide enhanced nodes with the ability to analyse this field, the processing functionalities were implemented as part of the forwarding decision, and therefore all the logic was included in a new forwarding strategy. The forwarding strategy in the NDN architecture determines whether, when, and where to forward an Interest packet. The proposed strategy is based on the well known best-route strategy (/localhost/nfd/strategy/best-route) provided as part of the NFD implementation [46]. Following this approach not only ensures backward compatibility but also allows the decision of which prefixes will support this novel functionality. The operation of the new strategy mainly differs from the original one in the process of satisfying an Interest, as shown in Figure 5a. Before satisfying an Interest, it is checked whether the optional *ApplicationParameters* field is present and the original data packet is processed accordingly, potentially generating a new data packet with the resulting information, as shown in Figure 5b. The producer should be aware of whether it is serving a CiC-enabled prefix; if that is the case it sends the aggregated content (i.e., disregard the *ApplicationParameters*) and offloads the processing to the network.

**Figure 6:**
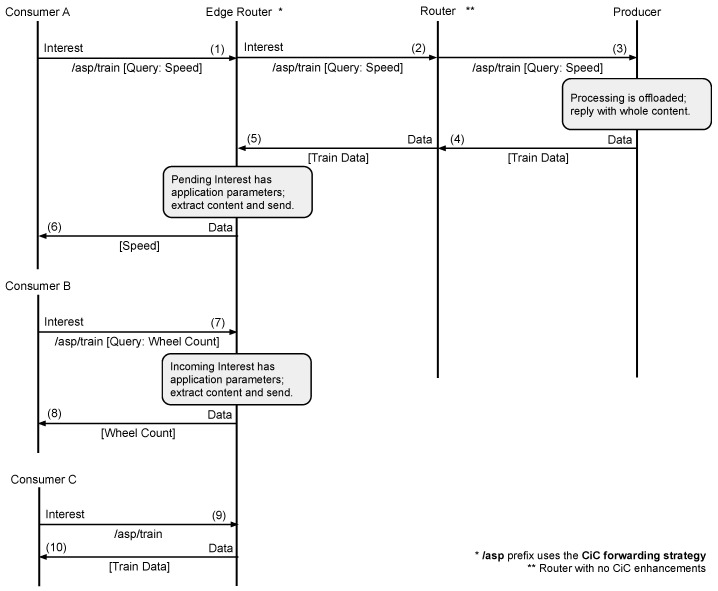
CiC Forwarding: message sequence diagram.

By following this design, the proposed mechanism inherently supports the request for a larger piece of information. By neglecting the *ApplicationParameters* field for CiC-enabled prefixes, the producer will reply with the data identified in the Interest name, leaving the content selection functionality to the edge networking entities. This approach is in line with the concepts sketched in Figure 3d. To better illustrate the operation of the proposed solution, Figure 6 shows the message sequence diagram that results from putting all the previous concepts together into a sample scenario. In this scenario, the /asp prefix is associated with the CiC forwarding strategy enabled at the *Edge Router*, while the *Router* node does not have CiC enhancements. First, *Consumer A* requests (Message 1) a piece of the *Train Data* content (i.e., just the train speed information) by including a query in the *ApplicationParameters* field. Since the content is not available at the intermediary content stores, the *Edge Router* and the *Router* forward the Interest towards the *Producer* (Messages 2–3). Since the producer knows that the prefix /asp is CiC-enabled, it replies with the complete *Train Data* content (Message 4), which is forwarded by the Router towards the *Edge Router* (Message 5). Before satisfying the pending Interest, the *Edge Router* checks whether it contains a query embedded in the *ApplicationParameters* field, processes the original content and generates a new data packet with the extracted information (Message 6). Subsequent requests (Messages 7 and 9) are analysed, and in the presence of a query, the same approach is followed, or otherwise, the complete content is provided.

To showcase the selective content retrieval, it is assumed that the content to be retrieved is in JSON format, which is a common format to provide structured IoT content. Notwithstanding, the main operational principles are generic and could be extended to other types of content. Accordingly, JSON Path [47], a query language for reducing and extracting JSON information, is used by the consumers to specify the desired pieces of content. This query is later executed by the enhanced routers to extract the desired information. The *jsoncons* (https://danielaparker.github.io/jsoncons/—(accessed on September 2022)) header-only library was used to implement the JSON-related functionalities in the developed prototype.

### 4.3. Security Considerations

Information security and privacy are major concerns when considering the deployment of any networking solution. Requirements such as access control, confidentiality, integrity, and authenticity are fundamental to fostering the wide adoption of any particular solution. This becomes particularly relevant when considering scenarios, such as the IoT, which tightly couple the physical and digital worlds [48]. Although ICN takes security into consideration by design [49], there is still a large set of requirements to be addressed to securely support the IoT [50].

In the particular context of the current proposal, there are other security challenges associated with the execution of in-network function execution for ICN that should be taken into account, as highlighted in Refs. [36,51]. The two major security shortcomings related to our work have to do with trust and access control.

Intermediary nodes are allowed to process cached data and produce “new” data. This breaks the original ICN signature verification process since the signer of the information will not be its producer. To address this issue we envision solutions relying on schematised trust [52] and the use of local trust anchors [53].

The possibility of selectively accessing individual pieces of content also challenges access control mechanisms. For example, in our scenario, while a particular consumer may not have access rights for the whole *Train Data* information, it may be allowed to check temperature-related data. As such, in addition to name-based access control [54,55], fine-grained access control policies regarding the content itself should be enforced.

However, these security-related mechanisms fall into general concerns of the NDN operation itself and as such, it should be the target of deeper efforts particularly focused on those aspects. For that reason, the proper design and enforcement of security mechanisms are left out of the scope of this paper.

## 5. Evaluation

The proposed solution promises to impact the retrieval of content in the following aspects: (i) reduce the traffic load by lowering the amount of information exchanged between the edge of the network and the information producers; (ii) better leverage cached content by extracting partial information from the content, when possible; and (iii) provide content processing at the network layer. Consequently, the evaluation targets these aspects by answering the following research questions:

(1) How does the proposed mechanism affect the amount of information exchanged between the edge of the network and the information producers?
  - Answered by analysing the number of hops followed by data packets before reaching the consumers.
(2) How does it impact the overall cache utilisation?
  - Answered by determining the space used for storing content in all the network content stores.
(3) How do applications perceive the effects of the mechanism?
  - Answered by determining the processing delay as observed by the applications (i.e., end-to-end application delay).

To analyse these metrics and to properly answer the corresponding research question, different network topology sizes should be taken into consideration. Additionally, to better assess the impact of the proposed solution in terms of cache utilisation, different freshness values should be considered. As such, a simulation-based evaluation better fits the assessment requirements. Accordingly, the developed proof-of-concept prototype was ported to the ndnSIM [56] simulation tool version 2.7.

### 5.1. Simulation Set-Up

**Figure 7:**
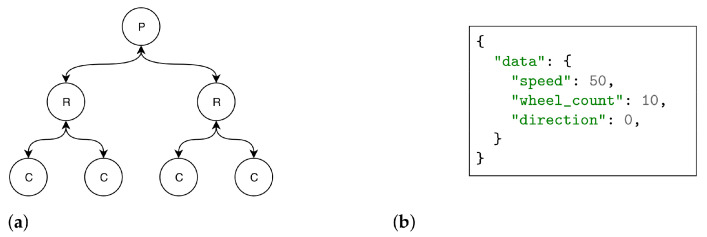
Simulation details: (**a**) Simulation topology (depth = 3); (**b**) JSON Train Data content.

The simulation scenario features a variable-depth binary tree topology. As represented in Figure 7a, the node at the root of the tree is the information producer, with the following layers being composed by NDN routers and the last layer by information consumers. The layer of routers closer to the consumers (i.e., edge layer) is provided with the CiC forwarding strategy. Consumers send randomly distributed Interests, during the 20 s of simulation, at a rate of 10 Interests/s. In addition, for every Interest, which query to be included in the Interest is randomly chosen (i.e., to request one of the following: (i) speed; (ii) direction; (iii) wheel_count; (iv) full content). The content generated by the producer is JSON-encoded information, as depicted in Figure 7b. Three different freshness values are considered: (i) 0 s—which means that content from a cache is never suitable; (ii) 50 s—which means that the content is cached once and remains suitable for the whole duration of the simulation; (iii) 10 s—which forces the cache to be refreshed once during the duration of the simulation. Table 1 summarises the key parameters used for setting up the simulation. To establish a baseline for comparison, a scenario following the same operational configuration, but with no CiC enhancements, was also simulated.

### 5.2. Results and Discussion

**Figure 8:**
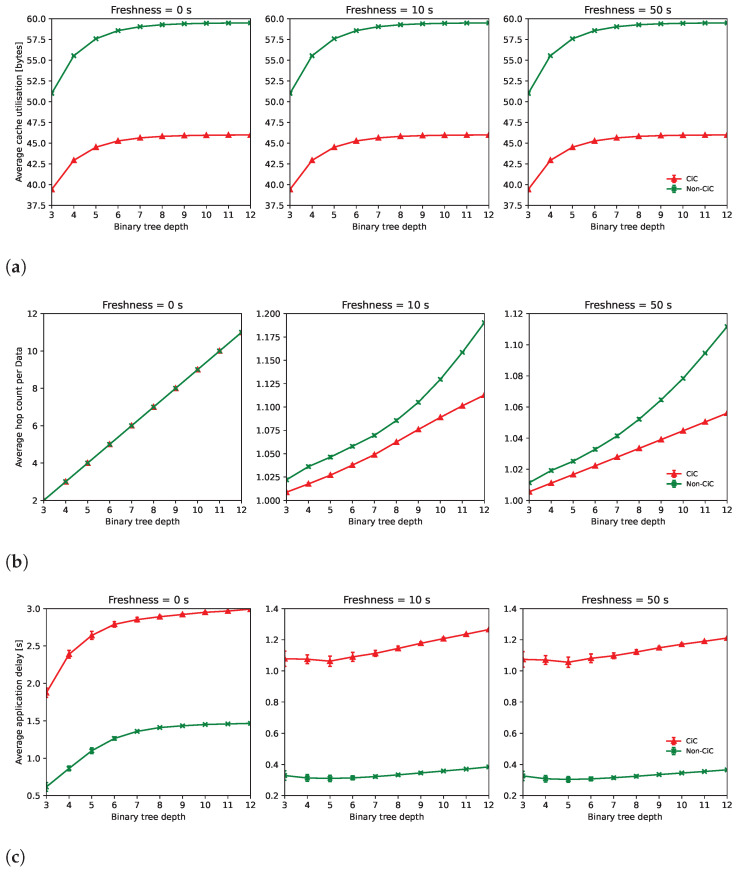
Evaluation results for different metrics and freshness values: (**a**) Average cache utilisation; (**b**) Average number of hops per data packet; (**c**) Average application level delay.

The simulation results are presented in Figure 8. As discussed earlier, three different metrics were evaluated, each answering one of the outlined research questions, and each of them calculated for different topology depths and data freshness values. The first one comprises the average cache utilisation (Figure 8a), which takes into account all the bytes inserted in each of the network content stores divided by the total number of caching nodes. The second refers to the average hop count followed by data packets (Figure 8b), calculated as the sum of the number of hops for each data packet received by the consumers divided by the total number of those data packets. The third considers the average application delay (Figure 8c), calculated by averaging the end-to-end application delay from all the issued requests.

As expected, the content store utilisation (Figure 8a) is larger in the non-CiC scenarios. This is due to the fact that the individual pieces of content are retrieved from the source and cached individually, in contrast with the CiC scenarios where only the complete *Train Data* content is exchanged over the network. The freshness has no impact on this metric because the updated content replaces the older content, but still, the amount of used space remains the same.

For the freshness value of 0, which means that the caching is completely neglected, it can be seen from Figure 8b that the average number of hops is roughly the same number of hops between the producer and the consumers (i.e., the depth of the binary tree minus 1). On the other hand, as soon as the cached content is suitable for retrieval, the CiC approach outperforms the base solution.

Finally, as expected, the application delay (Figure 8c) is larger on the CiC approach. The reason behind this is that while in the non-CiC approach the processing work of the producer is cached in individual packets, in the CiC approach the Edge Router has to perform this processing on every request. Notwithstanding, the CiC could be further extended to explore the trade-off between space and processing, allowing the Edge Router to cache the locally processed data and, as such, outperform the non-CiC approach (i.e., because of fewer hops and the consequent network delay).

It is important to highlight that, besides the savings in terms of network utilisation (average number of hops), the most critical impact of the proposed mechanism is that the processing is performed at the network itself, thus saving the precious resources from the possibly constrained IoT devices.

## 6. Conclusions and Future Directions

This paper discussed the ability for retrieving particular pieces of content from within other content, with particular emphasis on its application in an IoT scenario. Different strategies for realising such an approach were presented. A novel Content in Content forwarding strategy was designed and implemented, which enabled the network to extract the requested information from larger pieces of content, through the exploitation of the *ApplicationParameters* field of Interest packets. The developed proof-of-concept prototype was ported to the ndnSIM simulator to assess the impact of the proposed mechanism. For evaluation purposes, a use case scenario in which JSON encoded information is generated by sensors and partially retrieved by interested parties, was followed. The results showcase the suitability of the proposed approach.

Furthermore, we are now witnessing how digitalization efforts, such as Industry 4.0, have profoundly refactored how communications are being perceived by a myriad of different vertical sectors. As a result, such actors are, on the one hand, not only converting and integrating new ways and technologies for information inter-exchange into their businesses, but also, on the other hand, producing new utilisation and consolidation requirements towards such technologies. These requirements will help shape the next generations of IoT solutions, by not only incorporating the principles of newly established Internet architecture designs, but also by further extending them with new operational capabilities on their own. This stands as a great opportunity for more clean-slate oriented network designs to come forward, becoming established as a strong proposition towards the IoT value chain. As a result, such designs themselves will not only need to evolve considering incremental enhancements of their own key capabilities, but they will have to increasingly target higher degrees of flexibility that render them capable of being integrated in widely different application scenarios. This comes as a key addition to the already on-going efforts targeting interoperability between these new systems and well established architectures, such as IP [57].

Concretely, IoT composes heterogeneously rich environments, manifesting a plethora of available in-networking resources operating at cloud, edge, and fog levels, targeting improved QoS/QoE and resource efficiency [58]. The design produced in this paper contributed to such extensions by creating the basis for the utilization of NDN in the context of in-network computing and seamless orchestration of network micro-services at the edge for realizing increasingly complex and demanding IoT utilisation scenarios. In future works, we plan to extend this study by further analysing the highlighted trade-offs between caching and computation, as well as by considering different types of content, including unstructured IoT information.

## Figures and Tables

**Table 1:** Simulation set-up parameters.

Parameter	Value
Channel Data Rate	1 Mbps
Latency	10 ms
Interest Rate	10 Interests/s
CS Size	100 packets
CS Replacement Policy	LRU
Freshness	[0, 10, 50] s
Topology depth	(3, 12)
Simulation Duration	20 s

## Data Availability

Not applicable.
